# Do Integrated Community Psychiatry Services in Primary Health Care Settings Improve Continuity of Care? A Mixed-methods Study of Health Care Users’ Experiences in South Africa

**DOI:** 10.5334/ijic.7721

**Published:** 2024-07-18

**Authors:** Saira Abdulla, Lesley Robertson, Sherianne Kramer, Jane Goudge

**Affiliations:** 1 Centre for Health Policy, School of Public Health, University of Witwatersrand, Johannesburg, South Africa; 2 Department of Psychiatry, University of Witwatersrand, Johannesburg, South Africa; 3 Community Psychiatry, Sedibeng District Health Services, Sedibeng, South Africa

**Keywords:** integrated care, severe mental disorder, primary health care, community psychiatry, South Africa

## Abstract

**Background:**

A community psychiatry service is provided from selected primary health care (PHC) clinics in Gauteng, South Africa. This study described the demographic and clinical characteristics of health care users (HCUs), and explored HCUs’ experiences of these services in order to shed light on the challenges of integrating psychiatric services into PHC.

**Methods:**

A mixed-methods study was conducted at two PHC clinics, where 384 clinical records were reviewed and 23 HCUs were interviewed. In Clinic-1, community psychiatry services were co-located, while in Clinic-2, these services were physically integrated into the PHC clinic.

**Results:**

HCUs from both clinics were generally female (55%), had not completed secondary level education (65%), and were unemployed (80%). Both clinics struggled with medication stock-outs and had the same number of community psychiatry health care providers. Compared to the co-located clinic, the physically integrated clinic had insufficient consultation rooms (compromising confidentiality), higher caseloads (910 compared to 580), more HCUs with psychotic disorders (61% compared to 44%) and a history of missed medication (58% compared to 40%). In both clinics, overall care coordination was limited, although some nurses coordinated care for HCUs. While organisational integration approaches improved the proximity of mental health services, there were challenges in continuity of care within and across health care sites.

**Conclusion:**

Coordination and continuity of care were constrained in both clinics, regardless of the organisational integration approaches used. As low- and middle-income countries work towards integrating mental health care into PHC, the implementation of organisational integration approaches should consider physical space, caseload, HCU need, and the inclusion of dedicated providers to coordinate care.

## Introduction

Many low- and middle-income countries (LMICs) are committed to achieving universal health coverage, which ensures that all individuals receive the health services they need without suffering financial hardship [1]. This includes providing comprehensive care for people living with severe mental disorders (SMD) [2]. However, mental health care in LMICs is limited, with national surveys reporting treatment gaps of up to 80% [3]. In order to improve the accessibility of mental health services, a growing number of LMICs have begun integrating mental health into primary health care (PHC) services, as endorsed by the World Health Organisation (WHO) [4,5,6].

Integrated care is defined as the provision of a continuum of health care services based on the population’s health care needs, where people receive care that is ‘coordinated across the different levels and sites of care’ [7,8,9]. Integration aims to reduce challenges associated with vertical standalone services in order to facilitate access to comprehensive service delivery [10]. Organisational integration approaches vary from partial to fully integrated health system functions (including leadership, financial and human resources, and information systems), in order to deliver multiple services during a single visit (by one or different providers) [10]. However, approaches to achieving integrated care remains unstandardized, as its implementation has to be context specific and dependent on the availability of resources, population needs, and policy priorities [7].

In South Africa, mental health legislation and policy promote the integration of mental health services into PHC settings [11,12]. The policy supports the provision of high-quality, coordinated, recovery-oriented mental health care that is accessible within health care users (HCUs) local communities to improve HCUs’ well-being and functioning [11]. To achieve this, the policy endorses scaling integrated mental health services using a multidisciplinary team approach, training PHC and non-specialist providers in mental health service delivery, and improving continuity of care within and across health care systems [11]. These measures are intended to efficiently manage mental health disorders and other medical comorbidities, reduce institutionalisation, and strengthen health systems [11]. However, the policy does not provide guidance on how to implement integration, nor does it outline the mechanisms to enable continuity of care.

While some progress has been made in integrating mental health services in PHC in some provinces [11], efforts at integrating care have typically focused on mild to moderate mental disorders [13,14]. However, people living with SMD also need community-based care, if not more so than people living with milder disorders. SMD are more debilitating, are associated with complex multi-morbidity, and when left untreated often lead to repeated acute events that burden the health system and families [3,15,16]. In South Africa, it is estimated that 4% of the population are living with SMD [17].

In Gauteng province, community psychiatry services are located within selected PHC clinics to provide community-based care for people living with SMD [18]. Various organisational integration approaches are used to facilitate this care, despite uncertainties about how best to integrate mental health with PHC. Some clinics have co-located community psychiatry services on the same site but in separate buildings, maintaining separate operational systems, while others have physically integrated these services within PHC, sharing space, management and systems. This study aims to: 1) describe the demographic and clinical characteristics of HCUs accessing community psychiatry services to better understand the populations served, and 2) explore the experiences of people living with SMD in two PHC clinics (one physically integrated, the other co-located) in the Sedibeng District to shed light on the challenges of integrating care. The research questions addressed are: 1) What are the demographic and clinical characteristics of HCUs accessing community psychiatry services? 2) What are the key facilitators and barriers to integrating community psychiatry services in PHC settings, and how do these different integration approaches affect HCUs experiences of care?

### Background

The restructuring of the publicly-funded South Africa health system during the 1990s post-apartheid included the provision of free PHC services [19]. With the resulting increase in demand for care, natural population growth, the HIV and TB epidemics, and the social determinants of poor health such as unemployment [20,21], PHC clinics are operating beyond capacity. This is further exacerbated by inadequate infrastructure, resource and health care professional shortages, and insufficient funding [19]. Inadequate community-based care for people living with SMD including poor coordinated and continuous care were exposed in 2015/6, where vulnerable people living with SMD were moved from Life Esidimeni (a cluster of privately run medium care psychiatric hospitals in Gauteng province) to various non-governmental care homes served by PHC clinics to reduce costs [18,22]. Shortly after this relocation, 144 people died from various causes including starvation, trauma, and neglect [18].

Integrating community psychiatry services in PHC settings is intended to not only make services more accessible, but to also strengthen the continuity and quality of care [11]. There are three types of continuities of care [23]. Relational continuity refers to the ongoing relationship between the HCU and provider (or a consistent team of providers who collaborate to provide care) [23]. Information continuity refers to the availability and accessibility of HCUs’ clinical and treatment history data across various providers and between health care settings [23]. Management continuity refers to ensuring coordinated and consistent long-term care and follow-up [23,24,25]. Together these three forms of continuity enable a better understanding of an individual’s medical history and treatment preferences so that health care providers can deliver person-centred treatment plans that take into account the unique needs and goals of each HCU [26,27].

## Methods

This study used a mixed-methods approach in order to provide a comprehensive understanding of integrated care through triangulation. We conducted a retrospective record review to describe the demographic and clinical profiles of HCUs who accessed community psychiatry services in two PHC clinics in Sedibeng district, Gauteng province. Individual semi-structured qualitative interviews were conducted to explore HCUs’ experiences of care. Data were collected during the period between September 2021 and June 2022. The COVID-19 pandemic did not impact the study as lockdown regulations were relaxed in between the various waves, allowing data collection to proceed under normal service operations. This study is reported in accordance to the Standards for Reporting Qualitative Research (SRQR) guidelines [*Additional file 1*].

### Setting

Sedibeng district, an economically deprived peri-urban area in Gauteng province, covers a surface area of 4 173 square kilometres and has a population of approximately one million [20]. Approximately 4,500 community dwelling HCUs voluntarily access integrated community psychiatry services provided in 9 out of 36 PHC clinics in the district. This service is managed by the district mental health team, which includes psychiatric nurses, psychiatrists, medical doctors, psychologists, occupational therapists, and social workers.

### Study sites

Of the 9 clinics providing community psychiatry services, two were purposefully selected based on the catchment population and location of the clinics (Table 1). In the co-located clinic (Clinic-1), community psychiatry services are located in an outbuilding separate from the PHC facility and have separate management, human resources, and filing systems. In the physically integrated clinic (Clinic-2), community psychiatry services are provided in the same PHC facility, sharing the same space, management and filing system. This clinic has a significantly higher caseload (910 compared to 580 HCUs per month) but the same number of community psychiatry staff (Table 1).

**Table 1:** Description of community psychiatry services.


	CO-LOCATED CLINIC (CLINIC-1)	PHYSICALLY INTEGRATED CLINIC (CLINIC-2)

Catchment population	The clinic is located in a suburban area accessed by a racially mixed population of mixed socio-economic status.	The clinic is located in a township accessed by Black South Africans of poor socio-economic status.

Physical space	Separate outbuilding behind the PHC.	Back of the PHC clinic with limited space.

Service load	Approximately 580 HCUs.	Approximately 910 HCUs.

Filing systems (paper-based)	Separate clinical records and filing systems.	Shared clinical records and filing system.

Community psychiatry Staff	Community psychiatry services in both clinics have the same amount of allocated human resources who provide care to only mental HCUs: • Approximately five nurses are available daily on weekdays in each clinic. • One psychiatrist supervises both clinics for all age groups. • Each clinic has two to four doctors who provide psychiatric care once a week for adults, and once a week for children and adolescents. • Psychologists, social workers and occupational therapists provide individual therapeutic care bi-weekly.	

Physician roles	Psychiatric doctors provide outpatient psychiatric and general health care for uncomplicated medical comorbidities for HCUs with mental health conditions, whereas PHC doctors provide more complex physical health care for HCUs with mental health conditions and after-hours mental health care.	

Nurse roles	• Conduct mental health screening of new HCUs when they first access community psychiatry services. • Issue psychotropic medication and provide clinical follow-up for HCUs every month.	

	Focuses primarily on mental health.	Provides PHC nursing and mental health duties.

Down-referrals to PHC	Stable HCUs with SMD are down-referred to the mental health champion in the PHC clinic who issues repeat medication (that are prescribed by psychiatric doctors every six months).	

Follow-up	There is no formal system to follow-up with HCUs who do not attend their appointments.	


*Notes*: (1) Township refers to an area located on the outskirts of a city that was historically reserved for Black South Africans during the Apartheid era. (2) The racial categories used in this study are based on the South Africa government’s classification system used in official statistical reports [28].

In both clinics, stable HCUs are down-referred to satellite PHC clinics (including to the PHC service at the co-located clinic) where they collect repeat medication from “mental health champions”. Mental health champions are designated PHC nurses trained by the psychiatric nurses to advocate for mental health awareness in PHC clinics and help facilitate continuity of care.

### Retrospective record review

The quantitative data provides the demographic and clinical characteristics of HCUs accessing community psychiatry services, providing insights into the population served.

#### Data collection

Data were collected from the HCUs’ paper-based clinical records by the first author and two fieldworkers who were provided with standardised training based on the study protocol. Fieldworkers were also trained to collect data electronically using open source software (KoboToolbox™). The inclusion criteria, sampling process, and data collected are described in Table 2.

**Table 2:** Data collection methods.


METHOD	SAMPLING	INCLUSION CRITERIA	DATA COLLECTED

Retrospective Record Review	Systematic sampling method selecting every 3^rd^ clinical record. A total of 384 clinical records were selected (193 records from clinic-1, and 191 from clinic-2).	1)Age 18+ 2)Attended community psychiatry service at least once since their first admission to the clinic.	Demographics, mental and behavioural disorders, medical comorbidities, referrals, appointment and treatment adherence, and hospital admissions.

Semi-structured Interviews	Purposive sampling was used to select HCUs from the waiting room if they met the inclusion criteria. A total of 23 participants were interviewed (13 interviews clinic-1, and 10 interviews in clinic-2).	1)Age 18+ *2)Retained in care for a minimum of 24 months 3)Active clinic attendees (defined as having attended the psychiatric service at least once in the last 6 months), *4)Seen by PHC and/or allied health care providers, 5)Stable with no severe cognitive impairment, 6)Capacity to provide informed consent	Interview guide contained questions regarding the HCUs’ 1)Illness narratives, 2)Experiences of integrated care 3)Referral pathways, and 4)Treatment adherence.

Field notes	Using a pen-and-paper method, detailed field notes were taken each field day, including: • Clinic observations (physical layout, the flow of HCUs and staff, and interactions observed in waiting areas). • Observations of participants during interviews: mood, tone, non-verbal cues, and perceptions of the interviewee’s comfort level, hesitance, enthusiasm, etc. • Challenges and personal reflections on daily events to identify biases.		


*These criteria were included to gain rich insights into HCUs’ experiences of care.

#### Data analysis

Descriptive statistics using Chi-square analysis were used to describe the demographic and clinical characteristics of HCUs (including SMI diagnoses, comorbidities, treatment adherence, hospital admissions, retention in care, and referrals) by clinic using STATA 17 statistical software. Statistical significance was determined using p-values at the 5% level of significance.

### Semi-structured interviews

Qualitative interviews offer deeper insight into individuals’ experiences, feelings, and organisational contexts [29]. Understanding HCUs’ perspectives can help identify challenges in integrating care.

#### Data collection

The interviewer introduced the study to HCUs in the waiting room of the community psychiatry service. Interested HCUs were purposively selected for participation based on the eligibility criteria (see Table 2). Twelve pilot interviews (six per clinic) were conducted with participants in order to standardise the interview guide and improve interviewer techniques. These interviews were not analysed. Thereafter, a total of 23 interviews were conducted. In clinic-1, all interviews were conducted in English by the first author. In clinic-2, due to language barriers, a fieldworker conducted interviews in the participants’ preferred languages. Interviews were 15 to 60 minutes in duration excluding the consent process. Interviews were conducted in a private consultation room or a private space outside within the PHC grounds. Detailed field notes were compiled for every field day (see Table 2). Interviews were conducted until data saturation was reached, i.e., until no further themes or insights emerged. All digital recordings of the interviews were transcribed verbatim. Interviews that were not conducted in English were translated using a literal translation technique by the fieldworker.

#### Data Analysis

All anonymised interviews were manually analysed in accordance with Braun and Clarke’s [30] thematic analysis guidelines. Each author familiarized themselves with the data (transcribed interviews and field notes) and independently coded five of the same transcripts. Following this, we compiled and reviewed all initial codes during team meetings, grouping similar codes to form preliminary themes. The lead author then coded the remaining interviews, which were subsequently reviewed by all authors. Authors drew comparisons across sites and between participants in an iterative process of reflection. We collaboratively discussed emerging themes, resolving any discrepancies in interpretations through discussion until consensus was reached. Finally, we refined and named the themes, identifying clear examples from the data for each theme. Themes included infrastructure and resources, privacy and confidentiality, continuity with other health care institutions, continuity within clinics, continuity between physical and mental health care, and continuity with allied health care services. The use of multiple data sources (interviews, clinical records, and field notes) allowed for triangulation, improving the credibility and validity of the findings [31]. Clinical records were used to supplement missing information from interviews. For example, during the pilot interviews, we noted that some HCUs could not always recall specific details, such as the year they first accessed integrated care or the health care providers to whom they were referred. Participants’ characteristics and clinical profiles are presented in *Additional File 2*.

### Ethics

Ethical approval for this study was obtained from the University of the Witwatersrand, Human Research Ethics Committee and Sedibeng District Health Services. To ensure that HCUs had the capacity to provide consent to participate, the interviewer asked them to express, in their own words, their understandings of a) the purpose of the interview, b) the concepts of anonymity and confidentiality, c) the right to refuse to participate, and d) the right to withdraw at any time without consequence. HCUs that were able to do this were considered capable of providing informed consent. The mental health team was on standby during interviews, however, none of the participants displayed any signs of distress.

## Findings

### Demographic and clinical profiles of HCUs

In this section, we provide an overview of HCUs serviced in the clinics based on the retrospective record review. Overall, more than half (55%) of the HCUs were female, and nearly half (48%) were between the ages of 40–59 (Table 3). The majority of HCUs did not complete secondary level education (65%), and most (80%) were unemployed.

**Table 3:** Demographic profile of HCUs.


CATEGORY	CO-LOCATED CLINIC (CLINIC-1) *n* = 193		PHYSICALLY INTEGRATED CLINIC (CLINIC-2) *n* = 191		OVERALL *n* = 384	

	*n*	%	*n*	%	*n*	%

**Sex**						

Female	112	58.6	96	50.3	208	54.5

Male	79	41.4	95	49.7	174	45.6

**Age** (median: 45; range: 18–84 years)						

Adolescents (18–19)	2	1.0	2	1.1	4	1.1

Young adults (20–39)	54	28.1	62	32.9	116	30.4

Adults (40–59)	96	49.8	87	46	183	47.9

Older adults (60+)	41	21.2	38	20.2	79	20.6

**Highest level of education**						

None	5	2.8	3	1.8	8	2.3

Primary	23	12.9	41	24.1	64	18.4

Secondary	85	47.8	70	41.2	155	44.5

Matric (final school examination)	55	30.9	49	28.8	104	29.9

Tertiary	10	5.6	7	4.1	17	4.9

**Employment status**						

Employed	35	18.3	43	22.6	78	20.5

Unemployed	156	81.7	147	77.4	303	79.5


*Note*: Age groups reflect the categories used by the WHO for public health and policy making.

All HCUs had a SMD (Table 4). The co-located clinic had more HCUs with major depression (43%) and anxiety and stress related disorders (18%) compared to the physically integrated clinic (26% and 5%, respectively; p < 0.05). In contrast, the physically integrated clinic had more HCUs with psychotic disorders (61% compared to 44%; p < 0.05) and a history of missing their medication (58% compared to 40%; p < 0.05). Most HCUs (57%) were referred from the hospital to community psychiatry services in both clinics (p < 0.05).

**Table 4:** Clinical profile and referral pathway of HCUs.


CATEGORY	CO-LOCATED CLINIC (CLINIC-1) *n* = 193		PHYSICALLY INTEGRATED CLINIC (CLINIC-2) *n* = 191		OVERALL *n* = 384	

	*n*	%	*n*	%	*n*	%

**Mental and behavioural disorders**						

Anxiety and stress related disorders	35	18.1	9	4.7	44	11.5*

Major depression	83	43.0	50	26.2	133	34.6*

Bipolar and related disorders	53	27.5	64	33.5	117	30.5

Psychotic disorders	84	43.5	116	60.7	200	52.1*

**Medical comorbidities**	103	53.3	117	61.3	220	57.3

**Substance use**	113	58.5	109	57.1	222	57.8

**History of missing medication**	64	39.5	102	58.0	166	49.1*

**Psychiatric admissions**	139	72.0	144	75.4	283	73.7

Institution from which initial referral was made to community psychiatry*						

Hospital	80	50.3	116	63.4	196	57.3

Another PHC clinic	42	26.4	27	14.8	69	20.2

PHC in same clinic	25	15.7	30	16.4	55	16.1

Other (NGOs, schools, correctional services)	12	7.6	10	5.5	22	6.4

**Years accessing community psychiatry**						

<5 years	67	34.7	65	34.0	132	34.4

5–9 years	58	30.0	66	34.6	124	32.3

10+ years	68	35.2	60	31.4	128	33.3

**Referrals to allied health**	91	47.2	95	49.7	186	48.4


*Note*: *p < 0.05. (1) Anxiety and stress related disorders include post-traumatic stress disorder (PTSD) and panic disorders. Psychotic disorders include schizophrenia, schizoaffective disorder, and substance-induced psychosis. (2) History of missing medication refers to HCUs who had a history of missing their medication as noted by their providers on their clinical records.

### HCUs’ experiences of care

#### Infrastructure and resources

In both clinics, the community psychiatry services were conveniently located for HCUs: “*It’s within the community. I don’t travel long distance*” (P1-Clinic-2). However, HCUs complained about medication stock-outs, shortage of providers (especially mental health doctors and nurses), and inadequate facilities. Those attending the physically integrated clinic were further burdened by poor maintenance of the clinic: “*Sometimes the toilets are not functioning or dirty… there’s a JOJO (water storage) tank, but there’s no water*” (P8-Clinic-2). Better conditions in the co-located clinic appealed to users from surrounding areas: “*Some prefer [clinic-1] because in some of the other facilities…they’re not as equipped*” (P4-Clinic-1).

While the workload was greater in the physically integrated clinic (see Table 1), both clinics operated beyond capacity which resulted in long waiting times: “*The clinic is really small for all of us…we can’t be seen quickly because the sisters are short staffed…in mental health, we are a lot*” (P2-Clinic-1). In the physically integrated clinic, HCUs had to queue outside the facility regardless of the severity of their conditions: “*It is a very tough situation in this clinic only. You have to stand because you don’t have money to pay for the chair outside. I just want to sit down because when I meet the doctors, I am angry, my blood pressure is high… sugar is high. Can they just treat us well? We are begging because I can see the people here do not care about us”* (P5-Clinic-2). During the data collection, a strike disrupted clinical care at the physically integrated site: “*They turned us back at the gate. I had to come back after a few days for my medication*” (P7-Clinic-2). This affected some HCUs’ treatment adherence, as they were unable to afford transport costs to return to the clinic. In the co-located clinic, HCUs who missed their appointments or required care prior to their scheduled appointments were least prioritised by psychiatric doctors due to high caseloads: “*I’ve never ever sat by sister [nurse] and cried before… She [nurse] actually said to me she can’t do anything to my medication because only the doctor can do something for me because she’s can’t prescribe stuff like… so I just had to go another month… That’s how it is… you have to go on your date*” (P9-Clinic-1). While this did result in some HCUs adhering to their scheduled appointments, it also discouraged them from seeking care beyond their regular appointments, even if it was urgent: “*I can go back there but then they put you last in the queue*” (P7-Clinic-1).

#### Privacy and confidentiality

In the co-located clinic, there was sufficient space dedicated for the provision of mental health services. However, in the physically integrated clinic (Clinic-2), HCUs seeking care for the first time consulted with a psychiatric nurse in the corridor, while other HCUs received monthly follow-up care and repeat medication in an overcrowded room, compromising their right to privacy and confidentiality (See **Box 1**).

**Box 1 Poor privacy and confidentiality in Clinic-2**

HCUs waiting to see the psychiatric nurses are seated in the corridor. The psychiatric nurse, seated at a table in the middle of the corridor, calls new HCUs, one at a time, to conduct a history-taking interview. The interview includes sensitive questions about the HCU’s personal, medical and psychiatric history. The psychiatric nurse and HCU converse in hushed tones to prevent others from overhearing.

Other HCUs waiting to see the psychiatric nurses for monthly follow-up and medication collections are attended to in a room where 3–5 nurses perform administrative tasks and converse with each other. These nurses occasionally enter and exit the room while one nurse consults with the HCU.

(Fieldworker observations)

The limited number of consultation rooms meant that two psychiatric doctors worked in the same room, thus compromising confidentiality: “*Another patient comes in and sits down with another doctor in the same room. They talk while you are still with your doctor. I feel bad because they will be listening to what you are saying to your doctor. It’s not nice*” (P6-Clinic-2); “*Obviously you wouldn’t feel free to talk in this situation”* (P10-Clinic-2). Continued exposure to this arrangement seemed to lead to some desensitization: “*Well, I don’t take it seriously, we are used to that anyway. What can we do?*” (P10-Clinic-2).

#### Continuity with other health care institutions

The referral system linked HCUs from local general hospitals, remote psychiatric hospitals, private doctors and PHC to community psychiatry services. These referrals, often accompanied by letters containing the HCU’s clinical information, facilitated a degree of informational continuity across organizations. However, there was often poor management continuity in both clinics. Firstly, HCUs were not monitored by the referring provider (or any other staff) and the responsibility of accessing services post-discharge was placed upon HCUs: “*They [hospital providers] transferred me to the clinic, but they never called me to find out if I am taking my medication [after discharge] and check if came here [to the clinic]*” (P10- Clinic-2).

Secondly, some HCUs were not immediately referred to community psychiatry services once discharged from the hospital. One HCU was finally referred to community psychiatry services after multiple psychiatric admissions in different hospitals: “*2003 was the first time I was admitted. After a year, it went on again during my divorce…then again at the mall… I was admitted again and I stayed on my treatment for just for a few weeks. … They never called me to find out if I am taking my medication [but] they transferred me to come to the clinic*” (P10-Clinic-2).

Lastly, clinic staff sometimes overlooked referrals for the management of medical comorbidities: “*I was actually diagnosed with [high] cholesterol when I was in hospital. But then sister [the mental health champion at PHC] realized that nothing was done. She looked at my file and nearly had a heart attack. She said: ‘What is going on here?’ That’s when she drew the blood, and when it came back, she said to me: ‘Not one but two tablets for you’*” (P9-Clinic-1).

#### Continuity within the clinics

In both clinics, HCUs who experienced relational continuity with their mental health doctor, PHC doctor, or the mental health champion experienced improved communication over time: “*When you [first] meet up with him or her, you are afraid to talk with them. Once I am used to the doctor, I communicate whatever issue I have…I get to talk more, they ask me how I am doing, how is treatment?*” (P1-Clinic-2). Relational continuity enabled trusting relationships where the provider learned of the HCU’s circumstances and provided support: “*She [psychologist] is concerned about my well-being… we are working on it at this moment*” (P10-Clinic-1).

HCUs who consulted with different doctors felt frustrated with having to repeat themselves. While clinical records enabled information continuity, poor relational continuity had a negative impact on health outcomes: “*The doctor knows what medication I get but every time it’s a new doctor then he changes it, he sees things differently. I told them it works, and they must leave it like that, but then they lessened the medication and then I got sick again. If I get the right pills, then it takes another two months to adapt again*” (P7-Clinic-1).

#### Continuity between physical and mental health care

In the physically integrated clinic, some psychiatric nurses coordinated their appointments with occupational therapists in order to provide care on the same day. In the co-located setting, the mental health champion coordinated care for HCUs who were down-referred to PHC for medication collection: “*[PHC staff] don’t want to listen, they just give me a date on my card. When I go to sister (mental health champion) and I show her my card, she takes me with my file to the HIV doctor, and says: ‘Why must she come two times a month?’ Then [the doctor] will get his script ready for me”* (P8-Clinic-1). The mental health champion also linked HCUs to PHC providers: “*I came to collect my script, and I told the sister that I just want something for pain. She asked me: ‘But what’s wrong?’ That’s when I told her that my shoulder, my neck, and my hip [were hurting]. She took me to the [casualty] doctor who took the X-rays and stuff, and that’s when she [doctor] diagnosed me with arthritis and sent me to physiotherapy*” (P10-Clinic-1).

Despite these efforts, there were gaps in ensuring continuity of care to identify and manage medical comorbidities. For example, one HCU attended both the co-located community psychiatry and PHC services for several psychiatric and medical comorbidities, and was known by clinic staff to use substances intravenously. However, she was only diagnosed with HIV by chance when she accompanied a friend to an HIV testing site: “*When I got [back] this side [community psychiatry], the sisters couldn’t believe I had HIV because they know me. They said: ‘Why didn’t we find out?*” (P8-Clinic-1).

#### Continuity with allied health care services

In both clinics, HCUs had access to allied health care services via referrals: “*[The dietician] teach us what foods are good for us to eat… and now I don’t have constipation…It’s nice of them to send us to the dietician”* (P6-Clinic-1). However, some HCUs were not referred. One HCU struggled with her increased weight gain but was not referred to a dietician: “*I was smaller but since I started drinking it [medication], I’ve been gaining weight… I am always eating…but they [community psychiatry providers] don’t say anything*” (P8-Clinic-2).

Others faced challenges with communication and service delivery from allied services. One HCU had been waiting for three years to receive her glasses: “*I want to read the Bible, I can’t see… I tested three times, 2019, 2020, 2021… If you ask them [optometrist]: ‘Do you follow up on what happened with the first test?’ [They say]: ‘No…’*” (P6-Clinic-1). Another participant experienced conflicting information from two providers, affecting his adherence to medication: “*He [occupational therapist] said once I am stable, I will stop taking the pills. They [mental health providers] said that I need to continue taking my treatment…but I am only going to take it for about 6–8 months*” (P7-Clinic-2). Some HCUs were unaware of the allied services available: “*What occupational therapist? What social worker?*” (P7-Clinic-1).

## Discussion

This study described the demographic and clinical characteristics of HCUs accessing community psychiatry services, and explored their experiences of care in two clinics (physically integrated and co-located integration). Most HCUs were referred from hospitals to community psychiatry services for further management. However, despite their crucial role in delivering community-based care, both clinics struggled with medication stock-outs, limited human resources, and operated beyond capacity. Furthermore, the physically integrated clinic, which served a population of poor socioeconomic status, had insufficient consultation rooms, higher caseloads, and more HCUs with psychotic disorders and a history of missed medication. In contrast, the co-located clinic served HCUs with mixed socioeconomic status, and had more HCUs with major depression and anxiety disorders. In both clinics, overall care coordination was limited, although some nurses (including a mental health champion) coordinated care for HCUs. There were also challenges in ensuring continuity of care for HCUs within and across health care sites. These challenges reflect the broader systemic issues within integrated care systems, such as the capacity to handle complex cases, and maintaining quality of care when caseloads are high.

Although integrated care is recommended to bridge the mental health care gap, its implementation varies across countries and settings [32,33,34,35,36]. Some settings have integrated or co-located community psychiatry services in PHC clinics [33,37], while others have implemented reverse integration by providing PHC services in behavioural health care settings [36,38]. Globally, the scarcity of mental health specialists often leads to trained PHC providers delivering mental health care services [18,32,39]. Consequently, some integrated care models have adopted nurse-led approaches to integration [32,40,41]. For example, in Ethiopia, psychiatric nurses (trained by psychiatrists) provided clinical supervision to PHC doctors, reviewing their treatment plans and validating diagnoses, while PHC doctors prescribed psychotropic medications [42]. This approach improved clinical and social outcomes for HCUs [42]. Similarly, in rural Australia, a nurse-led mental health service was developed to meet the needs of a vulnerable local community [32,41]. A critical factor to the success of this programme was the integration of highly skilled nurses who were authorized to prescribe medication [32,41]. Reflecting on this success, our study highlights the challenges HCUs face in accessing care outside scheduled appointments with psychiatric doctors, largely due to high caseloads. Empowering nurses with prescribing capabilities could improve service delivery and reduce doctors’ workloads, as HCUs have monthly consultations with nurses for medication collection and follow-ups. In line with this approach, South Africa’s National Strategic Plan (2023–2028) aims to train professional nurses to prescribe psychotropic medications under a doctor’s supervision [43]. However, the success of this strategy hinges on ensuring sufficient human resources are available in integrated care settings to prevent overburdening the already stretched nursing staff.

Studies in LMICs have found that integrating care can benefit health systems by improving access to services, reducing costs by utilizing existing resources in PHC, reducing HCUs’ costs by providing care within their communities, and reducing stigma associated with accessing care [40,44,45,46]. However, limited resources and insufficient space are substantial barriers to integrating services in already overstretched PHC clinics in LMICs [47,48,49,50]. The United Nations Convention on the Rights of Persons with Disabilities mandates that governments provide quality mental health services that respect HCUs’ rights and dignity, and are affordable, accessible and confidential [27]. In the physically integrated setting, limited space breached HCUs’ rights to privacy and confidentiality during consultations. This made it difficult for HCUs to communicate their needs to providers. This ethical issue has similarly been found in other settings in South Africa (North West province) and Uganda [46,50]. These findings demonstrate the resource constraints within the health system, which are further compounded by efforts to integrate care in LMICs.

As an intended goal of integrated care, continuity of care has been shown to yield positive outcomes for HCUs with SMD. These include reduced symptom severity, improved quality of life and social function, reduced hospital admissions, improved provider-HCU relationships, and less fragmented care [51,52]. However, poor communication between providers hinder continuity of care. Our study, alongside similar research in Norway [53], Canada [54], South Africa [40,46], and Uganda [55], found poor continuity of care within and across different levels of the health system. To address this, HCUs in the Norway study recommended collaboration between providers, and relational continuity between HCUs and providers [53]. These findings highlight the need for clear policy implementation guidelines, improved referral pathways and communication systems, and enhanced inter-sectoral collaboration to strengthen integrated and continuity of care.

Successful integrated care also requires active participation by providers to facilitate coordination across services within clinics and between institutions. Some nurses coordinated care, i.e. psychiatric nurses in the physically integrated clinic and a mental health champion in the co-located clinic. The mental health champion’s effectiveness could be attributed to relational continuity, fostering trusting relationships with HCUs, and the champion’s values, attitudes, and dedication to care coordination. There were also notable distinctions between the champion and psychiatric nurses. The champion had a lower caseload and exclusively consulted with stable HCUs, while nurses managed a larger number of HCUs, including those with more complex conditions. HCUs with more complex conditions may benefit from enhanced personalized coordination of care, similar to the attentive support provided to stable HCUs by the mental health champion. This aligns with the principles of collaborative care, where case managers coordinate care among multidisciplinary teams and provide support to PHC providers [34,37]. Despite considerable literature on the coordination role that case managers in high income countries play [34,37], further research is needed to understand the potential role of case managers in more resource constrained settings in LMICs.

### Limitations

This study has several limitations. HCUs who were lost to follow-up were excluded, limiting insights into perceived barriers to accessing community psychiatry in PHC. Interviewers were outsiders to the community and were perceived to be in a position of authority by some HCUs. While the role of the interviewer was clarified to the participants, this power dynamic could have led to social desirability bias, albeit the critique and introspection from the HCUs suggest otherwise. Passers-by or clinic staff interrupted some interviews, disrupting the flow of the interviews. This made it difficult for participants to complete their sentences despite probes. This reflected the clinic’s operational challenges. Data from the retrospective record review may have been under-reported in both clinics: some records had missing information, others were lost and replaced, resulting in incomplete HCUs’ histories. In the co-location clinic, some HCUs had two separate files (for community psychiatry and PHC services), however only community psychiatry files were reviewed due to time constraints. This may have resulted in the under-reporting of medical comorbidities.

## Conclusion

Although integration improved access to mental health services, continuity and integrated care were constrained in both clinics, regardless of organisational integration approaches employed. In the physically integrated clinic, care was more integrated, but the quality was compromised due to the lack of space and the greater demand for care; in the co-located clinic, services were less integrated, however the quality of care was not compromised by inadequate space. It is therefore difficult to recommend a definitive organisational integration approach based solely on the results of this study.

As LMICs work towards integrating mental health care into PHC settings as recommended by the WHO [5], it becomes crucial to adapt organizational integration strategies to each unique context. Factors such as financial and human resources, physical space, caseload, and prevalence and types of SMDs should be considered prior to integrating care. Future efforts should focus on improving care coordination and continuity in both clinics to improve the overall quality of care, including assessing the feasibility of appointing dedicated health care providers to support integration.

## Additional Files

The additional files for this article can be found as follows:

10.5334/ijic.7721.s1 Additional file 1. Standards for Reporting Qualitative Research Checklist.

10.5334/ijic.7721.s2 Additional file 2. Characteristics of HCUs interviewed.
