# Supplementary material for: Do Integrated Community Psychiatry Services in Primary Health Care Settings Improve Continuity of Care? A Mixed-methods Study of Health Care Users’ Experiences in South Africa
Source: Int J Integr Care. 2024 Jul 18;24(3):8. doi: 10.5334/ijic.7721 (PMC11259117; doi:10.5334/ijic.7721)
Supplement: Additional file 1. — Standards for Reporting Qualitative Research Checklist. [file ijic-24-3-7721-s1.pdf]

Additional file 1. Standards for Reporting Qualitative Research Checklist

Topic	Description	Page number
1. Title	Concise description of the nature and topic of the study Identifying the study as qualitative or indicating the approach (e.g., ethnography, grounded theory) or data collection methods (e.g., interview, focus group) is recommended	1
2. Abstract	Summary of key elements of the study using the abstract format of the intended publication; typically includes background, purpose, methods, results, and conclusions	1
Introduction		
3. Problem formulation	Description and significance of the problem/phenomenon studied; review of relevant theory and empirical work; problem statement	2
4. Purpose or research question	Purpose of the study and specific objectives or questions	2
Methods		
5. Qualitative approach and research paradigm	Qualitative approach (e.g., ethnography, grounded theory, case study, phenomenology, narrative research) and guiding theory if appropriate; identifying the research paradigm is also recommended; rationale	5
6. Researcher characteristics and reflexivity	Researchers' characteristics that may influence the research, including personal attributes, qualifications/experience, relationship with participants, assumptions, and/or presuppositions; potential or actual interaction between researchers' characteristics and the research questions, approach, methods, results, and/or transferability	Declarations
7. Context	Setting/site and salient contextual factors; rationale	3
8. Sampling strategy	How and why research participants, documents, or events were selected; criteria for deciding when no further sampling was necessary (e.g., sampling saturation); rationale	5-6
9. Ethical issues pertaining to human subjects	Documentation of approval by an appropriate ethics review board and participant consent, or explanation for lack thereof; other confidentiality and data security issues	6
10. Data collection methods	Types of data collected; details of data collection procedures including (as appropriate) start and stop dates of data collection and analysis, iterative process, triangulation of sources/methods, and modification of procedures in response to evolving study findings; rationale	3
11. Data collection	Description of instruments (e.g., interview guides,	5-6

instruments and technologies	questionnaires) and devices (e.g., audio recorders) used for data collection; if/how the instrument(s) changed over the course of the study	
12. Units of study	Number and relevant characteristics of participants, documents, or events included in the study; level of participation (could be reported in results)	Additional file 2
13. Data processing	Methods for processing data prior to and during analysis, including transcription, data entry, data management and security, verification of data integrity, data coding, and anonymization/de-identification of excerpts	6
14. Data analysis	Process by which inferences, themes, etc., were identified and developed, including the researchers involved in data analysis; usually references a specific paradigm or approach; rationale	6
15. Techniques to enhance trustworthiness	Techniques to enhance trustworthiness and credibility of data analysis (e.g., member checking, audit trail, triangulation); rationale	6
Results		
16. Synthesis and interpretation	Main findings (e.g., interpretations, inferences, and themes); might include development of a theory or model, or integration with prior research or theory	6-11
17. Links to empirical data	Evidence (e.g., quotes, field notes, text excerpts, photographs) to substantiate analytic findings	8-11
Discussion		
18. Integration with prior work, implications, transferability, and contribution(s) to the field	Short summary of main findings; explanation of how findings and conclusions connect to, support, elaborate on, or challenge conclusions of earlier scholarship; discussion of scope of application/generalizability; identification of unique contribution(s) to scholarship in a discipline or field	11-13
19. Limitations	Trustworthiness and limitations of findings	13
Other		
20. Conflicts of interest	Potential sources of influence or perceived influence on study conduct and conclusions; how these were managed	Declarations
21. Funding	Sources of funding and other support; role of funders in data collection, interpretation, and reporting	Declarations
