# Supplementary material for: Do Integrated Community Psychiatry Services in Primary Health Care Settings Improve Continuity of Care? A Mixed-methods Study of Health Care Users’ Experiences in South Africa
Source: Int J Integr Care. 2024 Jul 18;24(3):8. doi: 10.5334/ijic.7721 (PMC11259117; doi:10.5334/ijic.7721)
Supplement: Additional file 2. — Characteristics of HCUs interviewed. [file ijic-24-3-7721-s2.pdf]

Additional file 2: Characteristics of HCUs interviewed

Participants	#Years accessing Clinic	Age	Gender	Education	Employment	Mental and behavioural disorders	Medical Comorbidities	Substance use	Hospital Admissions	Appointment defaults	Medication compliance
P1-C1	13	21	Male	Secondary	U/E	Depression, ADHD, Intellectual Disability, Severe Conduct disorder, Reactive attachment disorder, Oppositional defiant disorder	Epilepsy	-	0	No	Compliant
P2- C1	2	50	Female	Secondary	U/E	Major depression, Anxiety, Borderline personality disorder	Diabetes, Hypertension	-	0	No	Compliant
P3- C1	9	28	Male	Secondary	U/E	Schizophrenia, Substance-induced psychosis	-	Cannabis	+2	No	Compliant
P4- C1	5	32	Female	Tertiary	Employed	Bipolar Disorder	Epilepsy	-	2	Yes	Compliant
P5- C1	5	30	Female	Secondary	U/E	Bipolar Disorder; Intellectual disability	-	-	1	No	Compliant
P6- C1	12	58	Female	Primary	U/E	Major depression, Anxiety, PTSD	Hyperthyroidism, Arthritis	-	0	No	Compliant
P7- C1	16	47	Male	Secondary	U/E	Schizophrenia	Skin Cancer, High blood pressure	Alcohol, cannabis	2+	No	Compliant
P8- C1	9	46	Female	Secondary	U/E	Mood and psychotic disorder, Borderline Personality Disorder;	HIV, Hypertension, Hyperthyroidism, Epilepsy	Alcohol, crystal methamph etamine	0	Yes	Not always compliant
P9- C1	23	56	Female	Secondary	U/E	Major depression, GAD, PTSD	Asthma, Cholesterol, Epilepsy, Melanoma, Lupus	-	5	Yes	Not always compliant
P10- C1	13	51	Female	Secondary	U/E	Major Depression	High Blood Pressure Arthritis Chest pain Kidney-related infections	-	1	Yes	Not always compliant
P11- C1	6	40	Male	Tertiary	U/E	Bipolar mood disorder	-	Cannabis	6	Yes	Not always compliant
P12- C1	8	28	Female	Tertiary	U/E	Bipolar mood disorder	-	-		No	Not always compliant
P13- C1	11	30	Male	Secondary	U/E	Bipolar mood disorder; Substance-induced psychosis	-	Cannabis	10+	Yes	Not always compliant
P1- C2	2	53	Female	Secondary	Employed	Major depression with psychotic features	Hypertension	-	3	No	Not always compliant
P2- C2	12	61	Female	Secondary	U/E	Schizoaffective disorder	Renal impairment	-	4	Yes	Not always compliant
P3- C2	3	38	Female	Secondary	U/E	Major depression	HIV	-	1	No	Compliant
P4- C2	18	41	Male	Secondary	U/E	Bipolar mood disorder	Hypertension	-	1	Yes	Compliant
P5- C2	9	45	Female	Secondary	U/E	Major depression	Hypertension Diabetes	-	1	No	Compliant

P6- C2	13	44	Male	Secondary	U/E	Bipolar mood disorder	Hypertension	-	-	Yes	Not always compliant
P7- C2	2	19	Male	Secondary	U/E	Bipolar mood disorder; Substance-induced psychosis	-	Cannabis	3	Yes	Not always compliant
P8- C2	6	38	Female	Secondary	U/E	Schizophrenia	-	-	2	No	Not always compliant
P9- C2	6	29	Male	Secondary	U/E	Schizophrenia; Substance-induced psychosis	-	Cannabis, Alcohol	3	No	Compliant
P10- C2	11	61	Male	Secondary	U/E	Schizophrenia; Substance-induced psychosis	Hypertension	Alcohol	4	Yes	Not always compliant
